# Patient Experiences With a Remote Monitoring Pathway for COVID-19

**DOI:** 10.7759/cureus.26263

**Published:** 2022-06-23

**Authors:** Courtney Cheng, Karishma Manji, Lora Appel, Christopher Smith

**Affiliations:** 1 Internal Medicine, Royal College of Surgeons in Ireland, Dublin, IRL; 2 Family and Community Medicine, University of Toronto, Toronto, CAN; 3 Internal Medicine, OpenLab, University Health Network, Toronto, CAN; 4 Internal Medicine, Michael Garron Hospital, Toronto East Health Network, Toronto, CAN

**Keywords:** self-monitoring, digital health, virtual care, covid-19, remote monitoring

## Abstract

Introduction: Due to the coronavirus disease 2019 (COVID-19) pandemic, a remote monitoring pathway was developed at Michael Garron Hospital to allow individuals with confirmed or presumed COVID-19 infection to successfully manage their illness at home. This study aims to understand patients’ experiences on this remote monitoring pathway and to investigate the effectiveness of the pathway in preventing unnecessary emergency department (ED) visits and detecting severe infection.

Methods: A total of 35 semi-structured interviews were conducted over the phone. Researchers reviewed transcripts to come up with an index of nodes. Two researchers initially coded the same four transcripts to ensure high inter-rater reliability. The remaining 31 transcripts were coded by one researcher.

Results: Of patients, 80% (n = 28) had a positive experience on the pathway. Remote monitoring was effective in reassuring 22.9% of patients (n = 8) with mild-moderate symptoms that their symptoms were not significant enough to go to the ED and they were monitored at home. A total of 8.6% of patients (n = 3) were correctly identified as having severe symptoms while on the pathway and were asked to present to the hospital. For 8.6% of patients (n = 3), remote monitoring did not identify their severe COVID-19 illness. Of patients, 2.9% (n = 1) were incorrectly identified as having severe COVID-19 symptoms when they were clinically well.

Discussion: Remote monitoring is an effective tool to optimize healthcare resources during a pandemic. It reduces ER visits and provides a means for routine follow-up while minimizing virus exposure. Patients generally had a positive experience; however, more research needs to be done on optimizing the detection of severe infection.

## Introduction

The SARS-CoV-2 virus, which causes coronavirus disease 2019 (COVID-19), has spread worldwide since December 2019. As of April 4, 2021, there have been a cumulative number of 130,459,184 cases and 2,842,325 deaths reported globally [1]. Given the nature of transmission and high rate of severe respiratory disease and mortality [2], with a resultant shortage of personal protective equipment, hospital beds, personnel, and mechanical ventilators [3], a need for remote patient monitoring emerged at the forefront of national attention to provide care to patients with COVID-19 beyond the traditional in-person practice.

However, data on the use of remote monitoring tools to manage COVID-19 patients are sparse. At a healthcare system based in Minnesota, United States, a digital platform that was initially used for enhanced recovery after surgery was adapted to treat patients with COVID-19 symptoms [4]. This platform enrolled all patients who had been seen with potential COVID-19, but only 1% in this cohort ever tested positive, with the majority untested (92%) and the rest negative (7.2%). Thus, the applicability of this remote monitoring pathway to patients with confirmed COVID-19 is unclear. This study did report simple satisfaction feedback with the pathway, with a Net Promoter Score of 66.5%, but a response rate of only 9%, similarly limiting any generalizability [4]. Moreover, 4% of patients who activated the program visited the emergency department (ED), and although patients recounted avoiding ED visits due to the availability of a healthcare provider [4], further qualitative data from those patients who did return to ED should be collected to understand how the program can be improved.

A remote monitoring program was also developed at the University of Pennsylvania Health System, which involved daily text messaging check-ins twice and a dedicated team of clinicians, to monitor patients with COVID-19 at home and escalate to human care when necessary [5]. This program had a high Net Promoter Score of 80, but interestingly, 70 patients went to ED on their own, of which only 27 (39%) needed to be admitted [5]. On the other hand, there were 537 patients whose self-reported data necessitated escalation to clinical care, but only 90 (17%) visited ED [5]. An investigation into these patients’ experiences could further improve program efficiency and patient satisfaction, which could then be applied to the implementation of programs for other conditions.

At a hospital based in the UK, a virtual COVID-19 ward was established to allow early discharge of COVID-19 patients [6]. However, results found that out of the 38 patients who re-presented to ED, 28 were readmitted, of which 16 were readmitted for reasons related to deteriorating respiratory conditions, while the remaining 12 patients were readmitted for non-COVID-19-related reasons [6]. Further analysis into patients’ reasons for returning to ED, particularly those who were not readmitted, may provide useful information to improve the efficiency and effectiveness of hospital resource allocation. Similarly, at the Royal Berkshire Hospital in the UK, a virtual monitoring pathway for managing COVID-19 patients was established [7]. Of the 31 patients who returned to ED, 19 (61%) were readmitted, but analysis of patient experiences was limited.

Across Ontario, Canada, similar remote monitoring programs have been developed for COVID-19. At the Sunnybrook Health Sciences Centre in Toronto, Ontario, the COVID-19 Expansion to Outpatients (COVIDEO) program was created to provide continual support for outpatients diagnosed with COVID-19 through the Ontario Telemedicine Network virtual care platform [8]. However, given the urgency of the pandemic and the recent implementation of their program, data regarding patients’ satisfaction with the intervention have not been reported.

Likewise, a remote monitoring pathway (CovidCare) was developed at Michael Garron Hospital (MGH) to allow individuals with confirmed or presumed COVID-19 infection to successfully manage their illness at home. This pathway leveraged virtual care technologies to provide a clinical evaluation when needed through processes supported by clear escalation protocols based on patient self-entered data, nurse review, Roth score, and community-acquired pneumonia symptom (CAP-Sym) score. The goal was to avoid hospital visits that risk the spread of infection while providing reassurance to patients that they could be adequately cared for from home. Though there has been a pervasive interest in the use of remote patient monitoring for COVID-19 patients [9], little is known about patients’ experiences with this intervention, and additionally, the value of these programs in reducing unnecessary utilization of in-hospital care.

Objectives

Our objectives were to understand patients’ experiences on a COVID-19 remote monitoring pathway and to determine the effectiveness of the pathway in preventing unnecessary ED visits and detecting severe COVID-19 infection.

## Materials and methods

This was a qualitative study of 35 patients' semi-structured interviews comprising a combination of open- and closed-ended questions. Interviews were transcribed using transcription software and edited by two researchers (CC and KM) to ensure accuracy. Data were analyzed using NVivo software (QSR International, Melbourne, Australia) based on pre-agreed nodes.

Setting

This study was conducted at Michael Garron Hospital in Toronto, Ontario, and was approved by the Michael Garron Hospital Research Ethics Board (reference number: NR-286).

Participants

A purposive sample of all COVID-19-positive patients enrolled in CovidCare at MGH up to July 13, 2020, who either had medium- and high-level flags but did not return to ED and were successfully managed at home (Group A, n = 39), or who did return to ED but were not admitted (Group B, n = 14), were identified using electronic medical records. Medium- and high-level flag patients who did not return to ED and were successfully managed at home were defined as those who reported worsened symptoms since the last check-in, resulting in medium or high alerts in the Vivify Health platform (Plano, Texas) (as outlined in the “Self-Quarantine Check-in Pathway” protocol shown in Appendix 1) but had no ED visits, hospital admissions, or death, and were successfully discharged from the program.

CovidCare pathway

**Figure 1:**
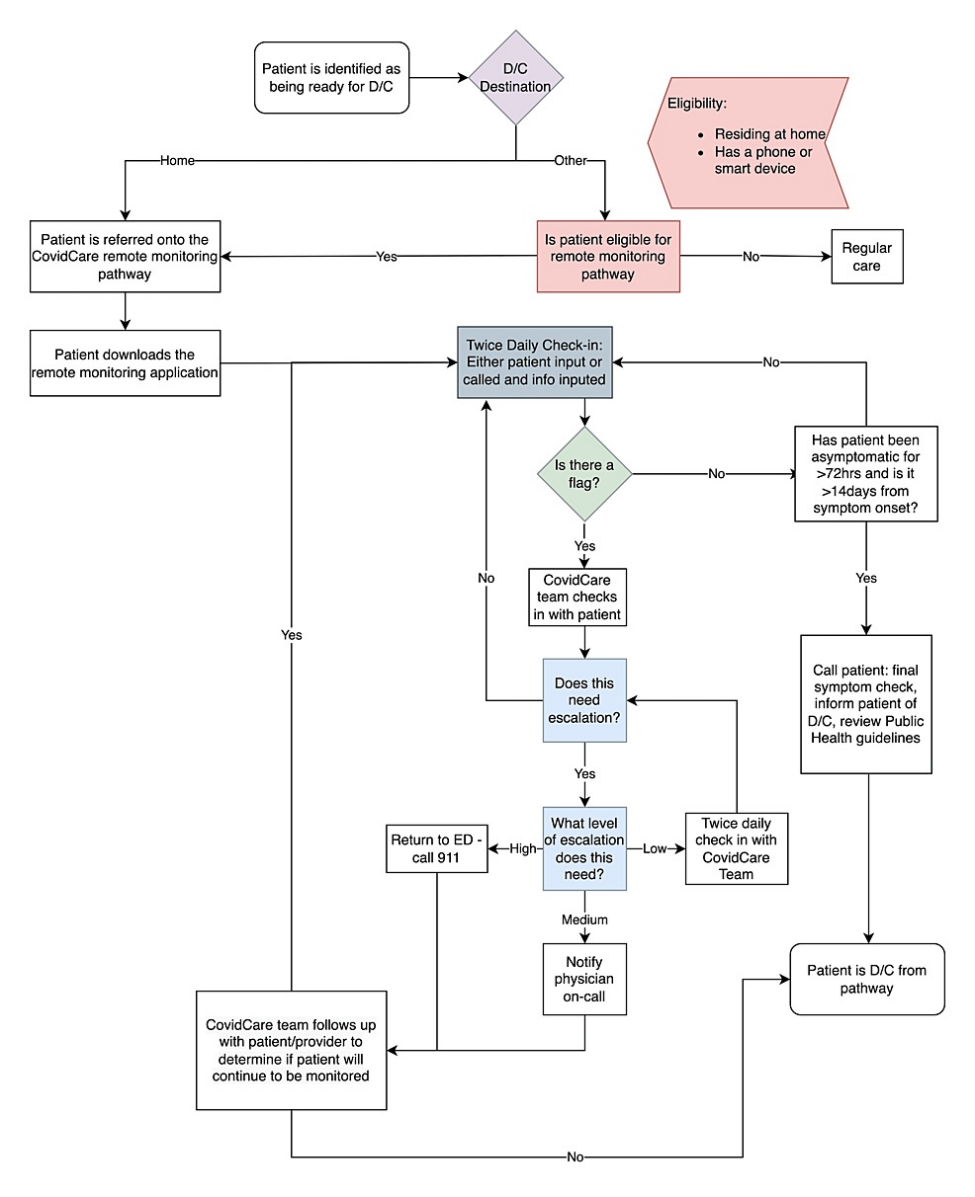
COVID-19 remote monitoring pathway

As shown in Figure 1, once patients meet the criteria for being discharged home, they are placed on the CovidCare pathway and guided through the process of downloading the Vivify Health application onto their device (e.g. smartphone and tablet). Through this application, they respond to questions about their symptoms twice a day. The check-in questions are included in Appendix 1. Patients who do not have access to a mobile phone or other smart devices receive twice-daily calls from nurses who ask about their symptoms and input the information into the remote monitoring system on their behalf.

If patients report new or worsening symptoms, a medium or high alert is generated in the system and the patient is followed-up by a nurse. If necessary, they can also be escalated to a family physician or specialist. On the other hand, if no alert is generated, the patient has been asymptomatic for over 72 hours, and it has been more than two weeks since the onset of their symptoms, the patient is discharged from the pathway. Otherwise, they continue to be monitored in the program.

Data collection

Data were collected in July 2020 using semi-structured interviews to elicit patients’ experiences with the remote monitoring pathway. Initial topic guides were based on questions in the Ontario Telemedicine Network “Patient Experience Discharge Survey” (Appendix 2). These preliminary topic guides were developed after broad consultation with experts in the field and revised in response to initial interviews. The final interview scripts are included in Appendices 3A and 3B.

Of the 52 patients who were phoned, 35 agreed to participate (Group A, n = 24; Group B, n = 11; response rate of 66%) and 34 provided oral consent to have the interview audio-recorded. Of those who declined an interview, reasons included lack of time and limited proficiency in English. Each patient was assigned a unique study number for de-identification purposes, and all data were stored on a password-protected USB and kept until the completion of the study. Based on saturation, a total of 35 in-depth interviews were conducted.

All interviews were carried out over the phone by a medical student researcher (CC) and involved a combination of open- and closed-ended questions. Interviews were conducted one-on-one with the patient or jointly with their caregiver as appropriate and lasted an average of 16 minutes.

Data analysis

Qualitative data from semi-structured interviews were analyzed using the grounded theory approach [10]. Interviews were audio-recorded, transcribed, manually checked for accuracy, and then coded and analyzed using NVivo 12 software.

Two researchers (CC and KM) reviewed two transcripts (24A and 64B) and used an open coding approach [11] to suggest a coding structure, which they consolidated into an initial framework. This framework was intended to comprehensively capture and categorize as much of the interview as possible, with the notion that only some codes and themes that directly address the research questions would be analyzed in greater detail later.

CC and KM then independently coded the interviews, suggesting additional nodes where relevant (e.g. “Patients in Group A stayed at home because they did not feel sick enough to return to ED” and “Usefulness of nurse check-ins and tracking”), and performed an intercoder reliability test in NVivo to determine the degree of agreement between the researchers. When the kappa coefficient did not meet the minimum acceptable value of 0.8 for intercoder reliability in qualitative research [12,13], CC and KM discussed and resolved the discrepancies and revised the coding framework accordingly (Appendix 4). To enhance the robustness of the analysis, an additional two interview transcripts (02A and 14B) were independently coded by both researchers using the updated framework, and a coding comparison query was performed again. As all kappa coefficients exceeded 0.8, indicating that CC and KM coded concepts and themes similarly, each researcher was assigned to code half of the remaining interviews.

For each of the four interviews that were initially coded by both researchers, all conflicts were resolved, and a combined document was produced that consolidated their coding. The same process (double coding and consolidating) was adopted for the one interview that was not audio-recorded (61), but for which detailed notes were taken during the interview.

After all interviews were coded, a numeric summary table was created to highlight the frequency that each node was mentioned by patients. Using the constant comparison method, findings relevant to four key themes were analyzed: (i) positive and negative aspects of the remote monitoring pathway, (ii) check-in frequency, (iii) patient suggestions, and (iv) sensitivity and specificity of the pathway.

## Results

The main themes that were analyzed include (i) positive and negative patient experiences related to the remote monitoring pathway, (ii) patient preferences regarding check-in frequency, (iii) patient suggestions to improve the pathway, and (iv) sensitivity and specificity of the pathway. A summary of our results is shown in Table 1.

**Table 1:** Summary of themes with corresponding sample quotes

Theme	Reasons	Mentioned by n = ?	Sample quote
Positive (n = 28, 80.0%)	Emotional support and care	30	“When you have this kind of monitoring program is like you are able to count on somebody” (43B)
	Program informative	16	“That's why I like, like all the information the what to do and what not to do, the Michael Garron nurses they provided me with that information” (15A)
	Staff level of knowledge	21	“They were really knowledgeable” (64B) “They were very knowledgeable” (57B) “It was actually very good idea to get in touch with those professionals so I won’t be biased based on my judgment or my assessment regarding myself” (15A)
	Respected and listened to	28	“They were very polite, very respectful. And they would always ask if it's the right time to call. Which I thought yeah they respected my space” (24A) “they didn't cut me off. I was allowed to talk as much as I needed to (...) They listened to me” (57B)
	Interaction with nurses	24	“Yes, having somebody who was a professional talk with me about it, I could ask (...) more and more questions about it. And I could talk through, you know, what I should do. And that was great (...) So having someone there to monitor me, keep tabs on me, to talk to, was helpful. I think it was helpful in my recovery”
Negative (n = 5, 14.3%)	Difficulty using the application	4	“I'm not used to using the app. My daughter had to do it for us” (23A)
	The app being available only in English and had directly through the application	1	“At the end, they hired a translator, it was translated to French and she could interact more easily” (33B)
	Difficulty contacting a nurse	1	“Tried calling you guys, but, you know, I kept getting voicemail and I would leave a message and you guys would never call me back. So I never bothered.” (CC65)
	Repetitive questions	1	“Well, it’s basically the same questions that you guys ask every day on the app.” (CC65)
	App not personalized	1	“There's nothing, you know, specific. I would feel it’d be better if you guys actually had like a video call or like a call from the actual thing instead of doing the app. That’s how I feel but I know you guys deal with a lot of patients as well.” (CC65)
Check-in frequency (n = 29, 82.9%)	>2 daily	1	“In between we don't know what really happened to the patient”
	2 daily	27	“If there was only 1 check-in, I would have felt a bit less comfort because those two calls really did help me. It was reassuring with the 2 calls. If there was only 1, I would have felt a bit more uncomfortable. With a second call, it was very…more comforting, knowing that at 5 pm or in the afternoon, a nurse would be calling. 2 was good, never felt the need for more than 2, 2 was more than enough”(61B)
	1 daily	1	“Once is fine” (65A)
	Customize frequency	3	“Switching to once a day once patient is stable” (39A)
Patient suggestions (n = 13, 37.1%)	Check-in questions	4	“I think if they check up on you three times that’ll be good” (CC32) “In between we don't know what really happened to the patient. That's why I think more time to call the monitoring patient to let that be know what really happened” (CC35) “Maybe they can take and consider switching to once a day once patient is stable” (CC39)
	Considering comorbidities	4	“I was having breathing issues...longer than COVID has been around“ (59A) “We took him to the hospital and they wouldn't even listen to the fact that he was had a problem with his chemotherapy...they assessed him as a COVID-19 patient and wouldn’t listen to anything else that we said” (23A)
	Language options	4	“if they can’t understand English, then they should have people who can help them. Because this city is full of people who are not completely comfortable with the English language” (23A)

Positive experience

The majority (n = 28, 80.0%) of participants expressed having an overall positive experience with the program and five patients (14.3%) had an overall neutral experience. The most frequently mentioned positive outcome was that the program provided emotional support and care (n = 30, 85.7%); one patient shared, “It was really a good program that I feel that someone is caring about me, someone is really thinking about me” (02A). Positive feedback included finding the program informative (n = 16, 45.7%), feeling comfortable with staff's level of knowledge (n = 21, 60.0%), and feeling listened to and respected (n = 28, 80.0%). The ability to interact with nurses was also mentioned by 68.6% (n = 24) of respondents, for example, one patient said, “yes, having somebody who was a professional talk with me about it, I could ask (...) more and more questions about it. And I could talk through, you know, what I should do. And that was great (...) So having someone there to monitor me, keep tabs on me, to talk to, was helpful. I think it was helpful in my recovery” (57B). Additionally, patients expressed an appreciation for being able to speak with the same nurses (n = 6, 17.1%), for example, one patient commented, “it was actually really nice to have consistent like I knew more or less who I was going to be speaking to in the evening and who in the morning. Which I think was helpful” (63B).

Negative experience

Five patients (14.3%) described an overall negative experience with the remote monitoring program. Several patients had difficulty using the application on their phones due to a lack of experience with smartphone applications, and one patient had concerns with the app being available only in English. Another patient had difficulty contacting a nurse directly through the application and felt questions on the pathway were repetitive and lacked personalization. Patients recommended a video call or phone call instead of the standardized check-in questionnaire using the Vivify Health application as a way to personalize the interaction, and they also stated they had left a voicemail that was not followed up on through the application.

Check-in frequency

The twice-daily check-ins were favorable among 27 patients (77.1%), and one patient would have appreciated more than two check-ins per day. Even among those who found the frequency adequate, two patients mentioned that once-daily check-ins would work for those who were improving, for example, one of the participants said, “for the first four days or five days after hospitalization, that's twice a day...and then after that, maybe it did not need to be twice a day for the full two weeks” (63B). A third patient mentioned that the frequency of check-ins would not be as important of a consideration for patients with milder symptoms, “for me it wouldn’t make that much difference but ... if the patient who had more severe symptoms, I think better be something really important in terms of once or twice daily check-ups” (15A).

Suggestions

Despite high levels of satisfaction, many participants (n = 13, 37.1%) offered suggestions on ways in which the pathway could be improved. The three most commonly reported areas of improvement included updating the check-in questions (n = 4, 11.4%), by including more signs and symptoms (02A and 57B). Additionally, they recommended increasing question specificity (63B and 65A) considering patients’ comorbidities (n = 4, 11.4%) such as sleep apnea, chemotherapy, and breathing issues pre-COVID-19. As well as offering more language options besides English (n = 4, 11.4%), for example, one patient reflected, “if they can’t understand English, then they should have people who can help them” (23A).

Sensitivity

The overall sensitivity of the pathway in detecting severe COVID-19 infection requiring assessment in ED was 50.0% (Appendix 5). Three patients (8.6%) were sent to the ED through the pathway and two of them received treatment with supplemental oxygen. There were three patients (8.6%) for whom the pathway did not detect their severe COVID-19 illness. In these cases, the patients themselves or a family member called emergency medical services and they were admitted to the hospital with COVID-19 symptoms. One of these patients felt there were not enough probing questions by the pathway on their diarrhea and vomiting symptoms to gauge the severity of their illness.

Specificity

The specificity of the pathway in detecting severe COVID-19 illness was 96% (Appendix 5). Eight patients (22.9%) felt reassured through the program that their symptoms were not significant enough to go to ED and did not have further clinical deterioration. There was one patient (2.9%) for whom the pathway suspected severe illness, but the patient was clinically well. This particular patient felt well but was sent to ED due to symptoms of shortness of breath; however, they were assessed and found to be well and advised to only return to ED if they felt sick.

## Discussion

This study aimed to analyze the effectiveness of a COVID-19 patient remote monitoring pathway implemented at MGH. This pathway was established to detect severe COVID-19 requiring assessment in the ED and to promote home monitoring of symptoms in milder cases to reduce unnecessary hospital visits. This study also aimed to understand patient experiences with remote monitoring, a field that has not been fully explored in the current literature [14].

The CovidCare pathway was effective at promoting home monitoring to reduce unnecessary ED visits. It empowered patients to manage their symptoms (n = 24, 68.6%) and educated them on how to reduce the transmission of COVID-19 (n = 14, 40.0%). Self-monitoring included breathing exercises, checking temperature, and using medication like Tylenol when appropriate. Prompts for self-monitoring were built into the Vivify application and reinforced by doctors and nurses. The pathway was effective in reducing unnecessary visits to the ED, as patients with mild disease felt reassured to continue at-home monitoring with close follow-up (n = 8, 22.9%).

The overall sensitivity of the pathway in detecting severe COVID-19 infection was 50%. The lack of objective data, specifically vital signs when assessing patients, made it difficult to evaluate patients who were unable to fully describe their symptoms. Specifically, one patient with vomiting and diarrhea was not sent to the ED but later presented with dehydration (CC57). Further characterization of the patient’s volume status through follow-up questions such as urine output and a full set of vitals, including blood pressure and heart rate, could have identified their clinically significant dehydration sooner. The lack of objective vital signs also decreased the specificity of the pathway. Assessors were more cautious to send patients to the ED for an assessment when they endorsed shortness of breath even if the patient had an alternative explanation for their symptoms (CC64).

Communication barriers that affected the sensitivity of the pathway were related to the fact that English was the sole language available on the application (n = 4, 11.4%), as well as a lack of open-ended questions (n = 2, 5.7%). Patients also had difficulty using a smartphone application for remote monitoring if they were not familiar with the technology (n = 4, 11.4%). Morgan et al. had similar concerns with technological literacy and language limitations in their text message-based COVID-19 remote monitoring pathway [5]. These barriers may pose a safety concern, especially if elderly patients underreport the severity of their symptoms.

Part of the challenge in detecting severe COVID-19 infection was that check-ins happened at two specific time points in the day. There was no notable way of predicting which patients who were well during these check-ins would clinically deteriorate at another time that day (CC31, CC61). Reviewing red flags for breathing with patients and families helped families make decisions about returning to ED when a nurse was not available to review the case, such as in the early morning hours (CC14). Another way to improve the detection of patients who may clinically deteriorate at another time during the day is by monitoring their vital signs remotely, including blood pressure, heart rate, oxygen saturation via pulse oximetry, and temperature [15]. These parameters could be monitored intermittently or continuously based on the patient’s risk of deterioration, which could then be relayed back to the technology platform overseen by the care team.

Consideration of patient comorbidities is another important step in increasing the sensitivity of the pathway. Patients with baseline shortness of breath (CC59) may require more specific questions to gauge whether changes in clinical status are related to COVID-19 or another underlying clinical condition. For example, patients with heart failure could be asked about pedal edema and changes in weight, which might be signs of shortness of breath related to heart failure rather than COVID-19. One patient on chemotherapy had panic attacks as a reaction to their treatment, and the family felt as though the COVIDCare pathway was ignoring other causes of shortness of breath until ultimately the patient was found to be COVID-19 negative (CC23).

In terms of overall patient experience, the pathway helped patients feel less lonely while they were self-isolating (n = 30, 85.7%). One patient described the power of this daily emotional support, which is a unique feature of the remote monitoring pathway, as being vital to their recovery.

Study limitations

Study limitations include a small sample size (n = 35), and the focus on a subpopulation of the Greater Toronto Area. Not all patients on the remote monitoring pathway participated in this study, highlighting possible selection bias for patients who had a positive experience on the pathway. Furthermore, since interviews were performed after patients’ experiences on the pathway, patients’ responses may have been influenced by their clinical outcomes. Interviews were conducted over the phone, which represented a limitation for patients who did not speak English, as there was a lack of non-verbal communication, and a family member may have been involved in translation (n = 2). Finally, this study did not have a control group of COVID-19-positive patients who were not placed on remote monitoring.

Future steps

Future versions of the remote monitoring pathway can include confirmation that the patient is comfortable using a smartphone application in English prior to enrollment. Patients who do not qualify can have the option to either have access to the application in other languages or have daily phone follow-up with a translator as needed. Access to at-home pulse oximeter monitoring and home blood pressure monitoring may also help increase the sensitivity of the pathway. A systematic review on COVID-19 remote monitoring by Vindrola-Padros et al. found mixed evidence for home pulse oximetry [14]. The notable limitations of home pulse oximetry were inconsistencies between different oximeter brands, lack of standardized patient education on how to monitor oxygen saturation at home, and underlying medical conditions such as chronic obstructive pulmonary disease (COPD), which may influence readings [14]. In contrast, O’Carroll et al., who are based out of Ireland, found that pulse oximetry monitoring was able to detect five patients (19.2%) who needed reassessment and out of those, four (15.4%) were admitted with worsening COVID-19 infection [16]. More robust data on oxygen saturation cut-offs while on remote monitoring and how they relate to clinical outcomes related to COVID-19 are needed [14].

Future versions of the pathway can also consider the personalization of questions based on patient comorbidities. For example, assessing baseline shortness of breath in patients with congestive heart failure or COPD may help prevent unnecessary ED visits. Offering open-ended questions and questions based on patient symptoms may help further clarify clinical status. For example, if a patient endorses diarrhea or vomiting, this can lead to questions about volume status, which can help with decision-making about going to ED.

Additionally, the pathway can be further customized based on the desires of the patient in terms of the frequency of check-ins. For example, nurses could check in with patients twice a day in the first week, and then patients could be given the option to continue with twice-daily check-ins or opt for once-daily check-ins for the second week. By doing so, patients who are recovering more rapidly and have less severe symptoms would not feel that the check-ins were redundant. Also, this feature would optimize resource allocation such that nurses would not have to unnecessarily follow up on patients who forgot to complete the twice-daily check-ins.

## Conclusions

In conclusion, we present a COVID-19 remote monitoring pathway that was established at MGH to manage COVID-19 patients. Overall, patients had a positive experience with the pathway, but further research is needed to improve the sensitivity of the pathway in detecting severe COVID-19 infection. We offer several ways to improve the patient experience as well as the efficiency of healthcare providers and resources, including prior assessment of patients’ comfort level with using technology, personalized questions based on underlying patient comorbidities, and customizing aspects of the pathway to align with patient preferences. Our findings can be used to improve the efficiency and utility of other remote monitoring programs, as remote monitoring becomes increasingly relied upon beyond the current pandemic.

## Figures and Tables

**Figure 2:**
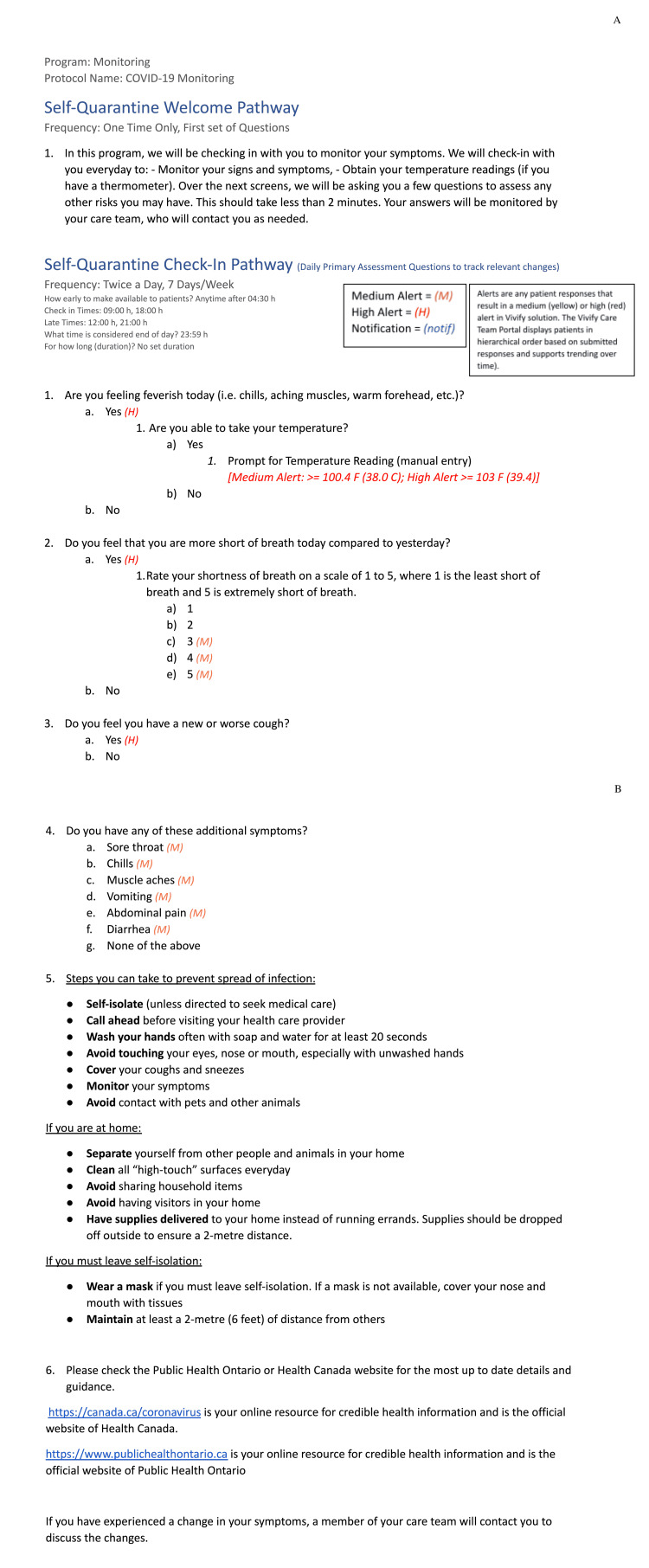
COVID-19 self-quarantine pathway

**Figure 3:**
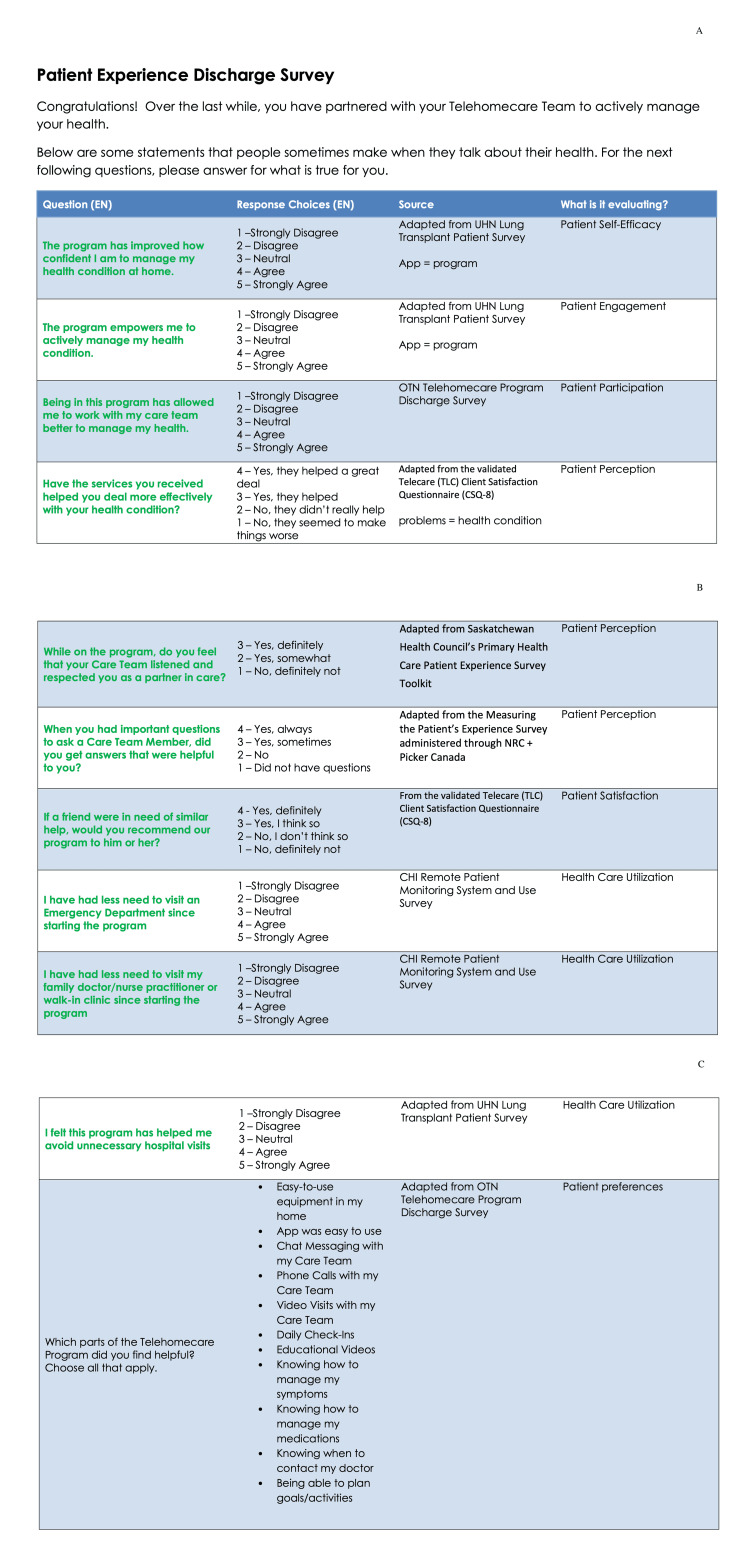
Patient experience discharge survey

**Figure 4:**
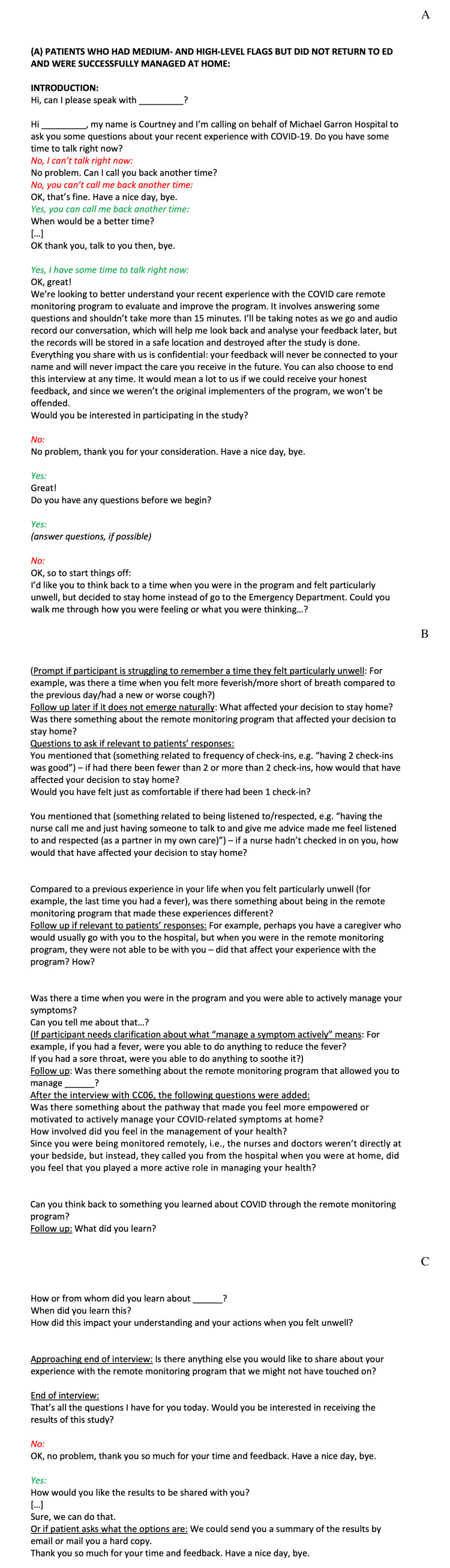
Group A interview script

**Figure 5:**
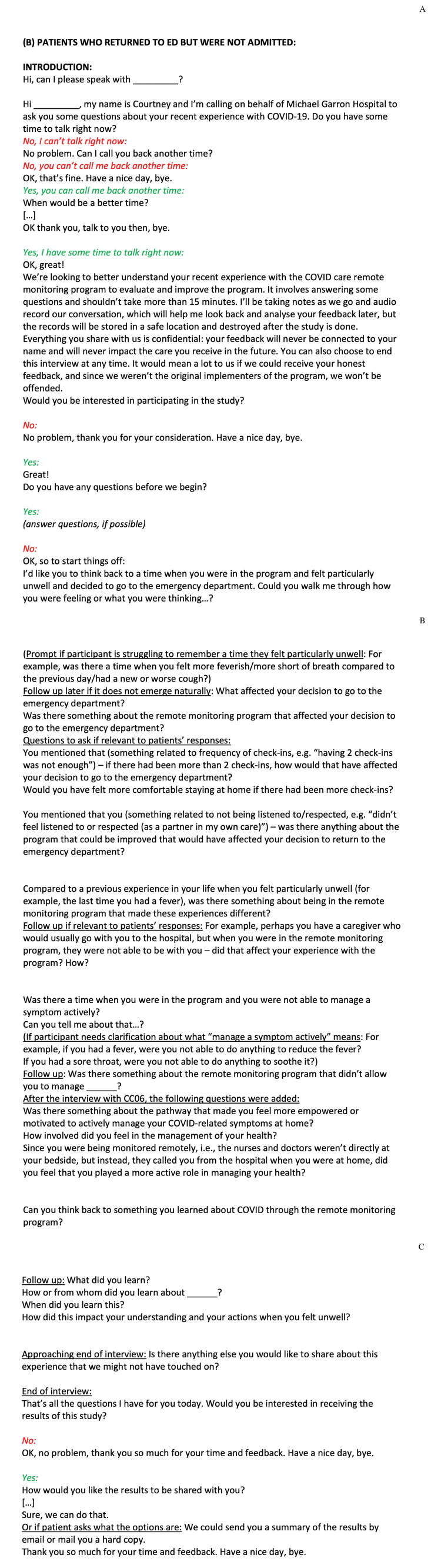
Group B interview script

**Figure 6:**
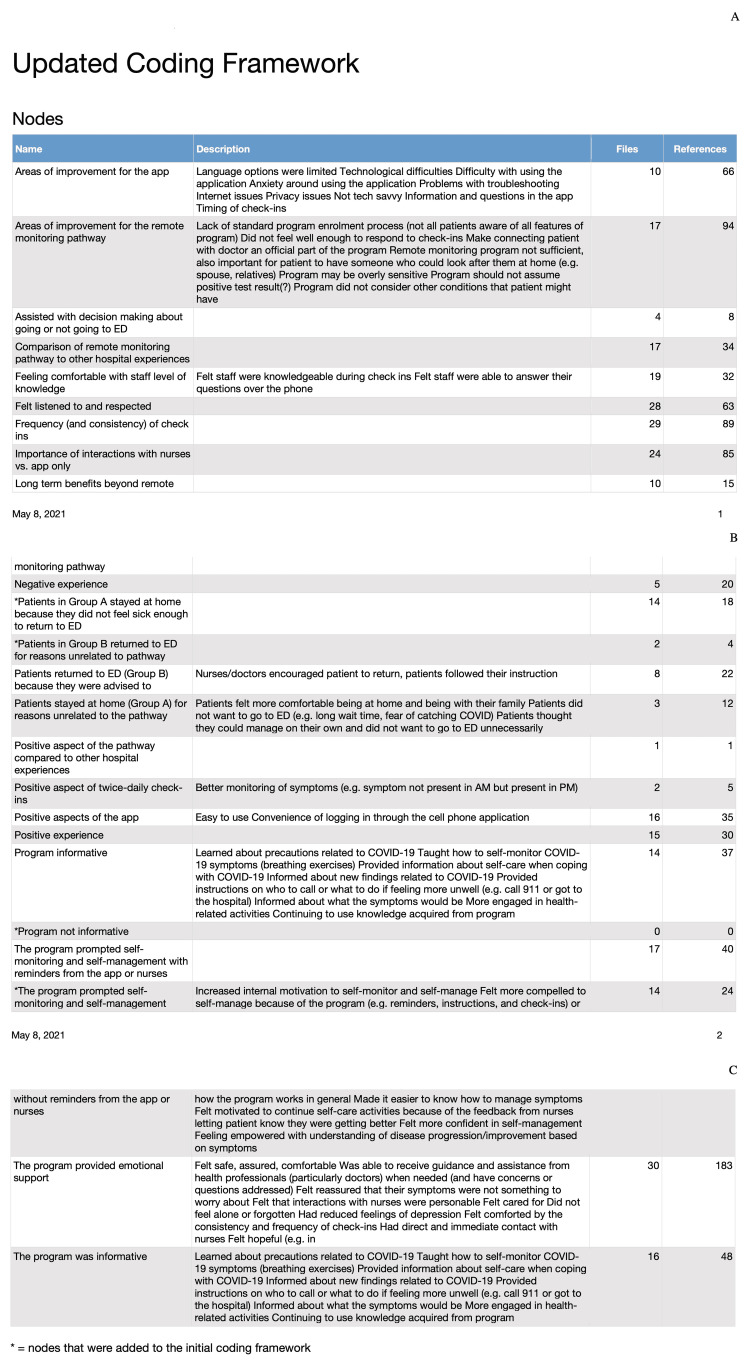
Updated coding framework

**Figure 7:**
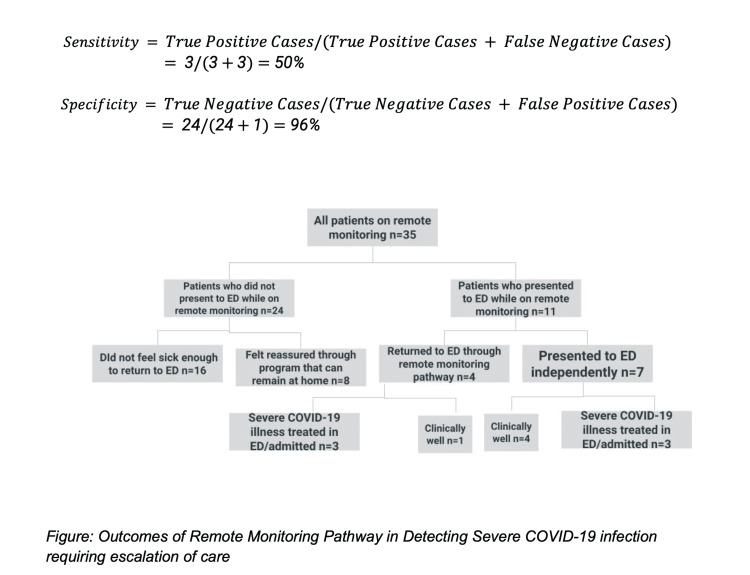
Calculating sensitivity and specificity of COVID-19 remote monitoring pathway

## Appendices

Appendix 1

Appendix 2

Appendix 3A

Appendix 3B

Appendix 4

Appendix 5
